# Impact of Fast SARS-CoV-2 Molecular Point-Of-Care Testing on Patients’ Length of Stay in an Emergency Department

**DOI:** 10.1128/spectrum.00636-22

**Published:** 2022-06-22

**Authors:** Audrey Baron, Olivier Peyrony, Maud Salmona, Nadia Mahjoub, Sami Ellouze, Maud Anastassiou, Constance Delaugerre, Jean-Paul Fontaine, Sylvie Chevret, Jerome LeGoff, Linda Feghoul

**Affiliations:** a Virology Department, AP-HP, Hôpital Saint Louis, Paris, France; b Emergency Department, Hôpital Saint Louis, Assistance Publique-Hôpitaux de Paris, Paris, France; c Université de Paris, INSERM, Equipe INSIGHT, U976, Paris, France; d Université de Paris, INSERM, U944, Paris, France; e Université de Paris, UMR 1153 CRESS, Biostatistics and Clinical Epidemiology Research Team, Paris, France; University of California, San Diego

**Keywords:** COVID-19, SARS-CoV-2, molecular detection, point of care, length of stay

## Abstract

The ID NOW COVID-19 system (IDNOW) is a point-of-care test (POCT) providing results within 15 min. We evaluated the impact of IDNOW use on patient length of stay (LOS) in an emergency department (ED). In the ED of Saint-Louis Hospital, Paris, France, adult patients requiring a rapid diagnosis of SARS-CoV-2 were tested with Cepheid Xpert Xpress SARS-CoV-2 or FilmArray respiratory panel RP2 in the virology laboratory between 18 October and 3 November 2020 (period 1) and with IDNOW between 4 November and 30 November 2020 (period 2). A total of 676 patients participated in the study, 337 during period 1 and 339 during period 2. The median LOS in ED was significantly higher in period 1 than in period 2 (276 versus 208 min, *P* < 0.0001). More patients spent less than 4 h in the ED in period 2 (61.3%) than in period 1 (38.3%) (*P* < 0.0001). By univariate analysis, factors associated with ED LOS were hypertension, anosmia/ageusia, number of patients per day, and ID NOW implementation in period 2. By multivariate analysis, the period of testing remained significantly associated with ED LOS. Rapid molecular SARS-CoV-2 POCT was associated with a reduced LOS for patients admitted to an ED.

**IMPORTANCE** During COVID-19 pandemic upsurges, emergency departments had to deal with a massive flow of incoming patients. The need for COVID-19 infection status determination before medical ward admission worsened ED overcrowding. The development of molecular point-of-care testing gave new opportunities for getting faster results of SARS-CoV-2 genome detection 24 h a day. In our study, we show, with a multivariate analysis, that the use of the POCT COVID-19 IDNOW reduced the ED length of stay by 1 h. The rate of patients who waited less than 4 h in the ED increased significantly. Our study highlights the benefit of COVID-19 molecular POCT for preventing ED overcrowding and facilitating bed allocation and SARS-CoV-2-infected patient isolation.

## INTRODUCTION

Severe acute respiratory syndrome coronavirus 2 (SARS-CoV-2), which causes coronavirus disease 2019 (COVID-19), has rapidly spread worldwide. Responsible for hundreds of millions of cases and millions of deaths, the COVID-19 pandemic has created an urgent and unprecedented need for large-scale diagnostic testing. Rapid detection of infected individuals is critical to decreasing SARS-CoV-2 transmission and optimizing patient care (1, 2). The reference standard for diagnosis relies on the detection of the SARS-CoV-2 genome by nucleic acid amplification testing (NAAT) on a nasopharyngeal sample (3, 4). Patient flow management and bed allocation are important elements in effective control of SARS-CoV-2 infection in hospitals and particularly in emergency departments (EDs) that can become rapidly overwhelmed (5). Usually with NAAT assays, samples are centralized in specialized virology laboratories, and the analysis itself may require several hours before results are released. Although antigen-based tests for SARS-CoV-2 provide a rapid result, their lower sensitivity may hamper optimal management in hospital settings (6, 7). Rapid molecular assays for point-of-care testing (POCT) of respiratory infections, mainly for influenza and respiratory syncytial viruses, have recently been developed to be used directly after sample collection in clinical wards. It enables optimization of the triage of suspected cases and isolation of more promptly confirmed cases compared to laboratory-based diagnostic methods and may thus reduce risks of virus transmission (8, 9). The use of POCT for influenza in the ED has been shown to reduce the length of stay (LOS) and improve patient bed management (10, 11).

The Abbott IDNOW COVID-19 assay is an isothermal nucleic acid amplification test that detects SARS-CoV-2 RNA directly from nasopharyngeal swabs within a turnaround time of 5 to 13 min. The IDNOW COVID-19 is CE marked and has a U.S. Food and Drug Administration (FDA) approval under an emergency use authorization (EUA) (12). The impact of its use of in an ED needs to be investigated. In this study, we evaluated the impact of Abbott IDNOW COVID-19 use on the length of stay of patients admitted to the ED of Saint-Louis Hospital, Paris, France.

## RESULTS

### Patients’ characteristics.

Between 18 October and 30 November, 2020, a total of 676 patients were enrolled, 337 in period 1 and 339 in period 2. The main characteristics of patients are summarized in Table 1. There was no statistical difference in demographic data of patients (sex and age) between the two periods. Regarding chronic comorbidities, we observed a significantly higher proportion of patients with chronic renal insufficiency in period 2 (5.0% versus 9.1%, *P* = 0.005) and a higher proportion of transplanted patients (hematopoietic stem cells transplantation or kidney transplantation) in period 1 (4.5% versus 1.5%, *P* = 0.021).

**TABLE 1:** Patients’ characteristics

Variable	Data for:		*P* value
	Period 1 (*n* = 337)	Period 2 (*n* = 339)	
No. of patients	337	339	
Sex (no. [%])			0.18
Male	191 (56.7)	210 (61.9)	
Female	146 (43.3)	129 (38.1)	
Age (median [IQR] [yrs])	62 [41–75]	61[46 –76]	0.56
Chronic comorbidities (no. [%])	176 (52.2)	155 (45.7)	0.11
HIV	13 (3.9)	12 (3.5)	0.84
Diabetes	48 (14.2)	55 (16.2)	0.52
Renal failure	17 (5.0)	31 (9.1)	0.05
Heart failure	11 (3.3)	8 (2.4)	0.50
Hypertension	80 (23.7)	93 (27.4)	0.29
Active cancer	54 (16.0)	73 (21.5)	0.08
Transplantation^*a*^	15 (4.5)	5 (1.5)	0.02
Obesity	8 (2.4)	7 (2.4)	1
SpO_2_ (median [IQR] [%])	100 [99–100]	100 [99–100]	0.1
Clinical symptomatology (no. [%])	138 (41.1)	144 (42.9)	0.70
Dyspnea	84 (24.9)	61 (18.0)	0.03
Asthenia	24 (7.1)	49 (14.5)	0.003
Sore throat	8 (2.4)	4 (1.2)	0.26
Ageusia/anosmia	4 (1.2)	3 (0.9)	0.72
Headache	23 (6.8)	17 (5.0)	0.33
Myalgia	22 (6.5)	17 (5.0)	0.41
Nausea/vomiting	19 (5.6)	29 (8.6)	0.18
Diarrhea	16 (4.8)	24 (7.1)	0.25
Cough	51 (15.1)	37 (10.9)	0.11
Fever	67 (19.9)	70 (20.6)	0.85
No. of symptoms per patient (no. [%])			0.93
1	36 (26.1)	44 (30.8)	
2	49 (35.5)	60 (42)	
3	34 (24.6)	27 (18.9)	
4	13 (9.4)	9 (6.3)	
5+	6 (4.3)	4 (2.8)	
Nurse triage level^*a*^ (no. [%])			0.096
1	1 (0.3)	1 (0.3)	
2	53 (15.9)	39 (11.7)	
3	132 (39.6)	129 (38.7)	
4	119 (35.7)	142 (42.6)	
5	21 (6.3)	21 (6.3)	
6	7 (2.1)	1 (0.3)	
Patients with positive test result (no. [%])	67 (19.9)	24 (7.1)	<0.0001
Rapid test indication (no. [%])			0.76
Suggestive symptoms of SARS-CoV-2	199 (59.1)	196 (57.8)	
No clinical symptoms	138 (40.9)	143 (42.2)	
Hospitalization rate			
Overall	189 (56.2)	243 (71.7)	<0.0001
Patients with SARS-CoV-2 positive results	33 (17.5)	17 (7.0)	0.0013
Time to results (median [IQR] [min])	261(207–339)	112 (69–159)	<0.0001
No. of patients per day (median [IQR])	81 (72.5–89.2)	61 (56.0–70.2)	0.0001
Hourly occupation rate (median [IQR])^*b*^	15.5 (14.0–17.2)	11 (10.0–12.0)	0.0004

a Data are missing for 4 patients in period 1 and 6 patients for period 2.

b Median number of patients present per hour.

The analysis of symptoms compatible with SARS-CoV-2 infection showed a higher proportion of patients with fatigue (7.1% versus 14.5%, *P* = 0.003) but a lower proportion of dyspnea (18% versus 24.9%, *P* = 0.03) during period 2. No significant difference in oxygen saturation was observed between the two periods. The frequency of patients with at least one symptom compatible with SARS-CoV-2 infection was similar between the two periods (41.1% versus 42.9%, *P* = 0.70).

At ED admission, there was no significant difference in the triage level between the two periods, suggesting a similar degree of clinical severity of patients (*P* = 0.096). No difference in SARS-CoV-2 rapid test indications was observed between the two periods (*P* = 0.76).

The percentage of positive SARS-CoV-2 tests was significantly higher in period 1 (19.9% versus 7.1%, *P* < 0.0001). A higher proportion of positive SARS-CoV-2 tests was also observed in the Paris area during period 1 (13.9% in period 1 versus 6.7% in period 2, *P* < 0.0001) (13).

### Hospitalization rate.

The overall hospitalization rate was significantly higher in period 2 than in period 1 (71.7% versus 56.2%, *P* < 0.0001). In contrast, the rate of SARS-CoV-2-positive patients among hospitalized patients was significantly higher in period 1 (17.5% versus 7.0%, *P* = 0.0013) (Table 1).

### Time to result.

**FIG 1:**
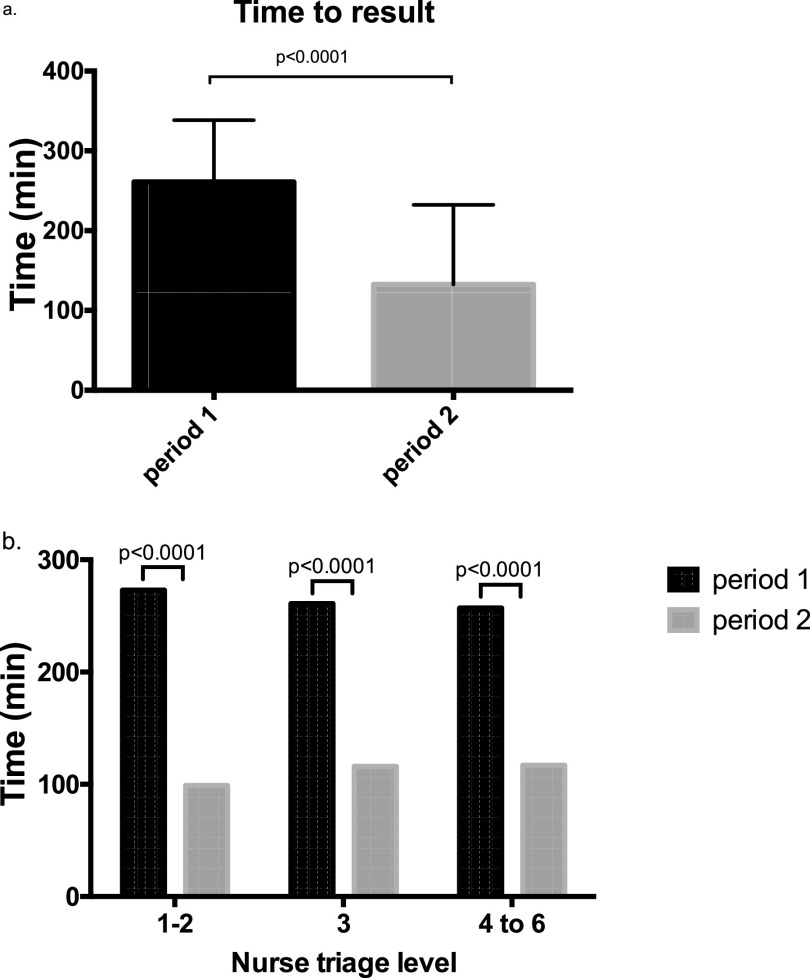
Time to results of SARS-CoV-2 detection. (a) Median time to result for period 1 (black bars) and for period 2 (gray bars). (b) Median time to result according to triage level. Triage level ranges from 1 (most severe) to 6 (less severe) (27).

The median time to result was significantly lower during period 2 (112 minutes; interquartile range [IQR], 69 to 159) than during period 1 (261 minutes; IQR, 207 to 339) (*P* < 0.0001) (Table 1). In addition, the time to result was significantly lower at each triage level during period 2 (Fig. 1). No significant difference in time to result was observed between day and night shifts for the two periods (Table 1).

### Patient flow.

The median number of patients per day was significantly higher in period 1 than in period 2 (81 [IQR, 72.5 to 89.2] versus 61 [IQR, 56 to 70.2], *P* = 0.0001), and the median number of patients per hour was significantly higher in period 1 than in period 2 (15.5 [IQR, 14.0 to 17.2] versus 11.0 [IQR, 10.0 to 12.0], *P* = 0.0004) (Table 1).

### Length of stay.

**FIG 2:**
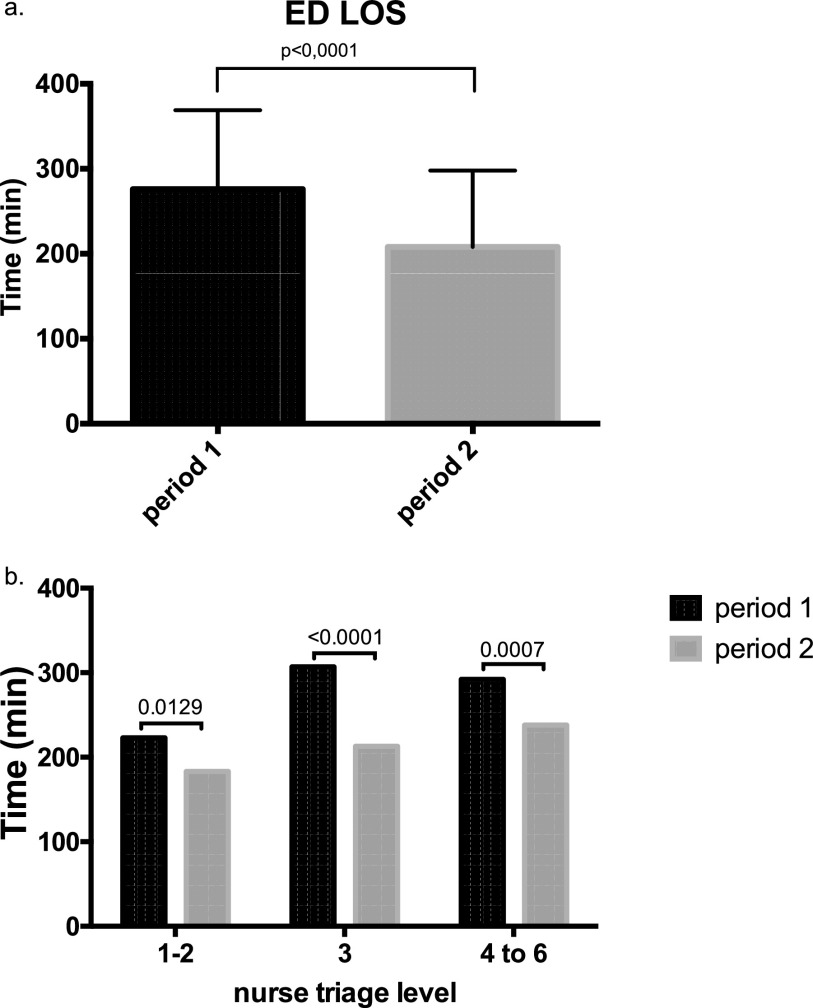
Patients’ length of stay in the ED. (a) Median time and interquartile range of patients’ length of stay in the ED, shown in black for period 1 and gray for period 2. (b) Median time according to nurse triage level.

The overall median ED LOS was significantly lower during period 2 (208 minutes; IQR, 136 to 296) than during period 1 (276 minutes; IQR, 195 to 369) (*P* < 0.0001) (Fig. 2a). At each triage level, the ED LOS was also significantly lower during period 2 (Fig. 2b). The percentage of patients who spent less than 4 h in the ED was significantly higher in period 2 than in period 1 (61.3% versus 38.3%, *P* < 0.0001). No difference in ED LOS was observed between day and night shifts for both periods (Table 1).

Univariate analysis showed that factors associated with ED LOS were history of hypertension (by increasing the average LOS by 32 min), anosmia/ageusia (by decreasing the LOS by 2 h), number of patients per day (by increasing the average LOS by 2 min), hourly occupancy rate (by increasing the average LOS by 9 min), and the period of the study (by decreasing the average LOS by 69 min) (Table 2). After adjusting for confounders in a multiple linear regression analysis, the effect of the period (corresponding to the effect of the IDNOW implementation) remained statistically significant, with an average decrease of 1 h in patients’ ED LOS in period 2 (Table 3).

**TABLE 2:** Univariate model for predicting the length of stay of patients in ED

Variable^*d*^	Estimate^*a*^	95% CI^*e*^	*P* value^*b*^
Age	0.06	−0.50 to 0.63	0.82
Sex	−8.97	−32.5 to 14.5	0.46
SARS-CoV-2-positive test	−1.47	−35.3 to 32.4	0.93
Rapid test indication comorbidities	−18.69	−42.1 to 4.70	0.12
HIV	25.83	−35.34 to 87.00	0.41
Diabetes	−4.26	−36.40 to 27.87	0.80
Renal failure	23.80	−21.13 to 68.73	0.30
Heart failure	−24.09	−93.95 to 45.76	0.50
**Hypertension**	**31.78**	**5.42 to 58.14**	**0.02**
Active cancer	−1.45	−31.02 to 28.11	0.92
Graft	39.10	−29.00 to 107.2	0.26
Obesity	0.18	−75.80 to 76.15	0.99
Clinical signs			
Dyspnea	−2.65	−30.78 to 25.49	0.85
Asthenia	23.13	−60.30 to 14.05	0.22
Sore throat	−73.93	−161.22 to 13.35	0.10
**Ageusia/anosmia**	**−120.307**	**−234.0 to −6.58**	**0.04**
Headache	−4.73	−53.68 to 44.21	0.85
Myalgia	−34.75	−84.21 to 14.72	0.17
Nausea/vomiting	4.27	−40.70 to 49.23	0.85
Diarrhea	23.38	−25.54 to 72.30	0.35
Cough	−9.18	−43.49 to 25.14	0.60
Fever	−4.19	−32.92 to 24.54	0.78
No. of symptoms	−4.96	−13.80 to 3.89	0.27
**No. of patients per day**	**1.67**	**0.79 to 2.55**	**<0.001**
**Hourly occupation rate**	**9.29**	**5.72 to 12.85**	**<0.001**
**Period** ^***b***^	**−69.39**	**−91.89 to −46.89**	**<0.001**
**Daytime** ^***c***^	0.36	−2.26 to 2.99	0.79

a Estimates are expressed in minutes.

b Period corresponds to the effect of the IDNOW implementation.

c Day shift (8:00 a.m. to 6:00 p.m.) versus night shift (06:00 p.m. to 08:00 a.m.).

d Data with significative differences are indicated in bold.

e CI, confidence interval.

**TABLE 3:** Multivariate model for predicting the length of stay of patients in ED^*a*^

Variable	Estimate	95% CI	*P* value
**Period**	**−64.78**	**−93.98 to −35.58**	**<0.001**
**Hypertension**	**33.37**	**7.79 to 58.95**	**0.01**
SARS-CoV-2-positive test	−22.95	−56.56 to 10.66	0.18
Ageusia/anosmia	−105.83	−216.8 to 5.15	0.06
**No. of patients no. per day**	**−2.12**	**−3.95 to −0.29**	**0.02**
**Hourly occupation rate**	**11.00**	**3.58 to 18.43**	**0.03**

a Data with significative differences are indicated in bold.

## DISCUSSION

The implementation of a molecular POCT test in the ED of our institution for detection of the SARS-CoV-2 genome was associated with a significant decrease in ED LOS by about 1 h. Despite differences in SARS-CoV-2 positivity, daily visits, and bed occupancy between the two periods, the impact of IDNOW use remained significant by multivariate analysis. Interestingly, the positive impact on LOS was observed for any level of triage, suggesting that rapid testing is valuable, whatever the symptomatology of patients admitted to the ED. With the decline in LOS, the number of patients staying less than 4 h in the ED increased 60% between the two periods. Discharge from ED within less than 4 h is considered a quality and performance indicator (14). These criteria are used by the National Health System in England to monitor patient management efficacy and have been associated with a significant reduction in overcrowding in EDs in England. Such an approach is of particular interest when heath systems’ capacities are overwhelmed in hospitals, as seen with the COVID-19 pandemic.

Previous works showed that rapid molecular assays for influenza virus detection (including IDNOW) performed directly in emergency departments could reduce ED LOS (10, 15). A recent study compared two periods in an ED, with and without the use of the IDNOW system for SARS-CoV-2 diagnosis (16). The results indicated, through a univariate analysis, that more patients were discharged within 6 h after ED arrival with the use of IDNOW. Our work confirmed, with a multivariate analysis, that a rapid molecular POCT had an impact on ED LOS.

Altogether, these studies emphasize that rapid detection of respiratory viral infections directly in emergency departments could improve patient management and bed occupancy. Reducing the time of infected individuals in emergency wards could also limit the risks of nosocomial transmission (15, 17, 18). A recent study using another molecular POCT showed that its implementation in the ED was associated with a significant reduction in the rate of hospital-acquired COVID-19 cases (19).

As expected, the time to results was lower with the use of IDNOW. This reflects the times requested for the transfer of nasopharyngeal samples to the virology laboratory and the longer technical times with the PCR tests used (50 min for GeneXpert and 45 min for FilmArray) than with IDNOW (5 to 13 min). The benefit of speed has also to be considered within the overall diagnosis performance. Indeed, the sensitivity of IDNOW is lower than real-time reverse transcription-PCR (RT-PCR), and its use could represent a risk for false-negative results and thus could hamper the isolation of infected individuals. To limit such a risk, testing was done at bedside and according to the manufacturer’s instructions without any dilution in viral transport media. Indeed, some studies reported a lack of sensitivity with nasopharyngeal swabs eluted in transport media (20–23). A recent study showed that the sensitivity of SARS-CoV-2 detection with IDNOW decreased from 98% when the test was performed with a dry swab to 62.5% when the swab was diluted into a viral transport media (24). However, this lack of sensitivity is observed for low viral loads. Smithgall et al. have shown that compared to RT-PCR, 100% of samples with cycle threshold (*C_T_*) values lower than 30 were detected with IDNOW (23). In contrast to *C_T_* values higher than 30, corresponding mainly to noncontagious or poorly contagious individuals, only 34.3% were detected with IDNOW (23). In our experience, we did not identify patients admitted to Saint-Louis medical wards after ED discharge that were found positive afterward (data not shown). In addition, for any suspicious case, nasopharyngeal samples could be tested with an RT-PCR assay in the virology laboratory.

The percentage of positive cases was significantly lower during period 2 at the time that the COVID-19 epidemic also slowed down in Paris. In contrast, the overall hospitalization rate was more important in period 2. This can be explained by the fact that non-COVID patients were reluctant to come to the hospital during period 1 for fear of being infected. Those patients waited for the epidemic to decline to come to the ED with deteriorated clinical situations requiring hospitalization more often. Any delay could contribute to conditions worsening, especially in our institution, specialized in the care of immunocompromised patients (cancer, hematological malignancies, transplantation). Further studies during and outside COVID-19 epidemic upsurges and in other clinical settings are warranted to confirm how rapid molecular POCT SARS-CoV-2 tests could improve the length of stay in ED.

### Conclusion.

Our study shows a significant impact of COVID-19 IDNOW use in an emergency department on the length of stay of patients. Our results highlight the potential benefit of very rapid screening of SARS-CoV-2 infection on patient management and bed occupancy.

## MATERIALS AND METHODS

### Objective.

The main objective was to compare the ED LOS of patients tested with a rapid molecular detection of SARS-CoV-2 in a centralized laboratory to that of patients tested with POCT IDNOW COVID-19 directly in the ED.

### Patients and study design.

We conducted a retrospective single-center study in the ED of Saint-Louis Hospital (Assistance Publique-Hôpitaux de Paris, Paris, France) between 18 October 2020 and 30 November 2020.

This period coincided with the second wave of the COVID-19 pandemic in France. The peak of positive new cases in France was reached on 7 November (25). We compared two periods, period 1, referred to as “pre-POCT,” and period 2, referred to as “POCT.” Period 1 started on 18 October and ended on 3 November 2020. Period 2 began with the implementation of IDNOW in the ED on 4 November. To compare a similar number of patients between the two periods, we stopped the analysis on 30 November 2020.

Adult patients who needed a rapid test for detection of SARS-CoV-2 virus by nasopharyngeal swabs were included in the study. Rapid tests in the ED were prescribed for patients who had symptoms suggestive of SARS-CoV-2 infection and who had to be admitted to a medical ward or for patients with an unscheduled surgery.

### Measurements.

During period 1, SARS-CoV-2 detection was performed in the virology laboratory with Cepheid Xpert Xpress SARS-CoV-2 (Cepheid, Sunnyvale, CA) or FilmArray respiratory panel (bioMérieux, Marcy l’Étoile, France). Tests were performed 24 h a day and 7 days a week from 8:00 a.m. to 6:00 p.m. by the virology laboratory technicians and from 6:00 p.m. to 8:00 a.m. by a laboratory medicine resident on call. Cepheid Xpert Xpress SARS-CoV-2 assay is a real-time reverse transcription-PCR (RT-PCR) test targeting N2 and E SARS-CoV-2 genes, and it provides results within 50 min. FilmArray respiratory panel 2.1 plus is a multiplex PCR assay for simultaneous detection of 20 pathogens (viruses and bacteria), including SARS-CoV-2. Results are available within 45 min.

During period 2, all samples for initial screening for SARS-CoV-2 infection were tested with POCT IDNOW directly in the ED. All nurses and physicians were trained for IDNOW use under the supervision of virology laboratory staff. Nasopharyngeal swabs were collected and tested directly, without any dilution in viral transport medium, on the IDNOW instrument according to the manufacturer’s instructions. The Abbott IDNOW COVID-19 system uses the nicking and extension amplification reaction (NEAR) technique, a rapid exponential amplification of short RNA sequences at a constant temperature coupled with a fluorescence-based detection. This assay targets the RNA-dependent RNA polymerase (RdRp) gene segment of SARS-CoV-2 and provides results in 5 to 13 min (26).

### Data collection.

All data were retrospectively collected using ED reports. This included demographic data (age and sex), comorbidities (HIV status, diabetes, hypertension, cancer, graft, obesity, chronic renal insufficiency, chronic cardiac insufficiency), the time of arrival to ED, the time of departure from ED, triage level (at ED arrival, a triage nurse assesses patient severity on a scale ranging from 1 to 6, with 1 being the most severe) (27), clinical signs related to COVID-19 (fever, dyspnea, cough, myalgia, anosmia/ageusia, headaches, fatigue, sore throat, diarrhea, nausea, and vomiting), and pulse oximetry (SpO_2_). We also collected the number of patients per day and the hourly occupancy rate (median number of patients present per hour) in the ED during the study period.

### Study outcomes.

The primary outcome was ED LOS defined as the interval between patient registration in ED and patient discharge from ED. Patients were either discharged home or admitted in a medical ward. The secondary outcome was time to result, defined as the interval between patient ED registration and SARS-CoV-2 test result release.

### Ethics.

Data analyses were conducted using an anonymized database. According to the French Health Public Law (CSP Art L 1121-1.1), such protocol was exempted from informed consent application. The study was approved by the Institutional Review Board of the French-Speaking Society for Respiratory Medicine, Société de Pneumologie de Langue Française (number CEPRO 2020-014).

### Statistical analysis.

Summary statistics (median and interquartile range, percentages) were tabulated to describe the distributions. Wilcoxon or Fisher’s exact tests were used to test for differences between groups. A time series model was used to describe the temporal evolution of the average LOS in the ED on each day before and after the introduction of the test. In order to adjust for potential confounders such as the number of daily ED visits, the hourly occupancy rate, or other variables associated with patients’ LOS, a multivariate linear regression model was used to estimate the effect of the introduction of the test on ED LOS on individual data. Statistical analyses were performed with SAS v9.3 (SAS, Cary, NC, USA) and R v2.13.0 (https://www.r-project.org/). All tests were two-sided, with *P* values of 0.05 denoting statistical significance.
